# Impact on Outcomes across KDIGO-2012 AKI Criteria According to Baseline Renal Function

**DOI:** 10.3390/jcm8091323

**Published:** 2019-08-28

**Authors:** Isabel Acosta-Ochoa, Juan Bustamante-Munguira, Alicia Mendiluce-Herrero, Jesús Bustamante-Bustamante, Armando Coca-Rojo

**Affiliations:** 1 Department of Nephrology, Hospital Clinico Universitario, 47003 Valladolid, Spain; 2 Department of Cardiac Surgery, Hospital Clinico Universitario, 47003 Valladolid, Spain; 3 Department of Medicine, Dermatology and Toxicology, School of Medicine, University of Valladolid, 47005 Valladolid, Spain

**Keywords:** acute kidney injury, chronic kidney disease, AKI staging

## Abstract

Acute kidney injury (AKI) and Chronic Kidney Disease (CKD) are global health problems. The pathophysiology of acute-on-chronic kidney disease (AoCKD) is not well understood. We aimed to study clinical outcomes in patients with previous normal (pure acute kidney injury; P-AKI) or impaired kidney function (AoCKD) across the 2012 Kidney Disease Improving Global Outcomes (KDIGO) AKI classification. We performed a retrospective study of patients with AKI, divided into P-AKI and AoCKD groups, evaluating clinical and epidemiological features, distribution across KDIGO-2012 criteria, in-hospital mortality and need for dialysis. One thousand, two hundred and sixty-nine subjects were included. AoCKD individuals were older and had higher comorbidity. P-AKI individuals fulfilled more often the serum creatinine (SCr) ≥ 3.0× criterion in AKI-Stage3, AoCKD subjects reached SCr ≥ 4.0 mg/dL criterion more frequently. AKI severity was associated with in-hospital mortality independently of baseline renal function. AoCKD subjects presented higher mortality when fulfilling AKI-Stage1 criteria or SCr ≥ 3.0× criterion within AKI-Stage3. The relationship between mortality and associated risk factors, such as the net increase of SCr or AoCKD status, fluctuated depending on AKI stage and stage criteria sub-strata. AoCKD patients that fulfil SCr increment rate criteria may be exposed to more severe insults, possibly explaining the higher mortality. AoCKD may constitute a unique clinical syndrome. Adequate staging criteria may help prompt diagnosis and administration of appropriate therapy.

## 1. Introduction

Acute kidney injury (AKI) is a global public health problem [1]. Using the 2012 Kidney Disease: Improving Global Outcomes AKI definition (KDIGO-2012) [2], one in five adults and one in three children worldwide experience AKI during a hospital episode of care [3]. AKI implicates a great burden in morbidity and mortality, increases sanitary costs [4,5], and affects long-term outcomes, including cardiovascular events and survival [6,7,8,9]. It is a clinical syndrome with a variety of aetiologies [10], once instituted, the treatment is mostly supportive [11,12], and the best approach remains prevention [13,14,15]. Based on the KDIGO definition of Chronic Kidney Disease (CKD) [16] its prevalence approximates 8–16% worldwide [17], affecting one in nine Americans and more than 300 million persons globally [18]. AKI is more prevalent in (and a significant risk factor for) patients with impaired renal function [2]; AKI, in turn, may act as a promoter of progression of the underlying CKD [2,19,20,21,22,23].

This evidence has led to a renewed interest in an old clinical concept: Acute on chronic renal failure, coined by Lim et al. in 1969 [24] and currently referred to as acute on chronic kidney disease (AoCKD) and its pathophysiology [23,25,26,27,28,29]. Few studies compare patients directly with prior normal (pure acute kidney injury [P-AKI]) and impaired renal function (AoCKD) during an AKI episode: Some conclude that patients with previous CKD bare worst clinical and renal outcomes [30,31,32,33,34], while others conclude that it could be protective against the negative consequences of AKI [35,36,37,38].

The most frequently used AKI classifications: RIFLE [39], AKIN [40] and KDIGO-2012 [2] do not discriminate patients with or without previous CKD, so the same criteria are used interchangeably in these individuals; therefore, a knowledge gap exists in the evaluation and staging of AoCKD. We hypothesized that AKI affects individuals with baseline normal and impaired renal function in a different way; in order to verify this theory we examined the distribution of patients in the strata defined by KDIGO-2012 criteria, and the relationship of AKI severity with short-term outcomes, such as in-hospital mortality and initiation of renal replacement therapy (RRT) between P-AKI and AoCKD subjects.

## 2. Experimental Section

All consecutive hospitalized patients treated by nephrologists in a 762-bed teaching institution, with a diagnosis of AKI by KDIGO-2012 criteria (Table 1), during a three-year period (June 2012 through May 2015) were reviewed.

**Figure 1:**
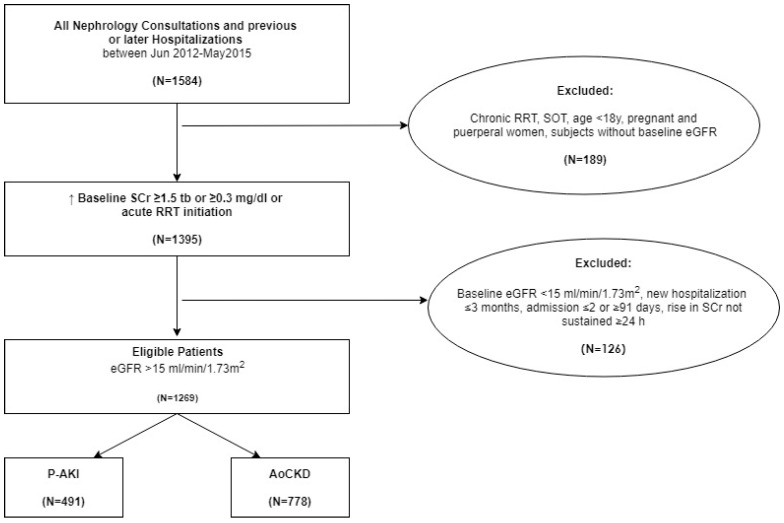
Study flow chart for inclusion and exclusion criteria. RRT: Renal replacement therapy; SOT: Solid organ transplantation; Tb: Times baseline. SCr: Serum creatinine; eGFR: Estimated glomerular filtration rate; P-AKI: Pure acute kidney injury; AoCKD: Acute on chronic kidney disease.

Inclusion criteria: Age ≥ 18 years, admission for >2 or and ≤91 days and rise in serum creatinine (SCr) sustained at least for 24 h. Exclusion criteria: History of solid organ transplantation, hospital readmission less than 3 months before or after index hospitalization, patients without baseline SCr, end-stage renal disease (previous RRT) or estimated glomerular filtration rate (eGFR) < 15 mL/min/1.73 m^2^ calculated by the four item Modification of Diet in Renal Disease formula (MDRD-4) [41] and pregnant and puerperal women (Figure 1). Previous and later hospitalizations were searched even if a nephrologist was not consulted and included as a new index hospitalization if they met the inclusion criteria.

We designed a retrospective cohorts study. The study conforms to the STROBE statement for reporting observational studies. Patients were divided in two groups: P-AKI (baseline eGFR ≥60 mL/min/1.73 m^2^) and AoCKD (baseline eGFR ≥15 and <60 mL/min/1.73 m^2^ for more than three months) (16). We defined baseline SCr as the lowest value in the six months prior to hospitalization, and when it was not available, we searched the 12 previous months [42]. Community-acquired AKI was defined as a SCr ≥ 1.5× increment at hospital admission [43]. We used the KDIGO-2012 stage associated with the peak SCr reached during hospitalization.

We registered several epidemiological and clinical features, intensive care unit (ICU) admission and hospitalization in medical or surgical units. The study was conducted in accordance with the Declaration of Helsinki, and the protocol was approved by the Ethics Committee of Área de Salud Valladolid Este (CINV 14–45); because of the anonymous and non-interventional nature of the study, they waived the need for informed consent.

Our primary objective was to compare the rate of in-hospital mortality across every criterion of the KDIGO-2012 AKI classification between groups. Our secondary objectives included comparing the rate of initiation of RRT, length of hospital stay (LOS), time to nephrology consultation, and dialysis-dependence at discharge in both groups.

Patient demographics are summarized using mean and standard deviations (SD) or median (25th–75th percentile) for continuous variables and counts with percentages for binary variables, as appropriate and according to data distribution. Normal distribution of data was analyzed using a Kolmogorov-Smirnov test. Continuous data was analyzed using Mann-Whitney U tests (between P-AKI and AoCKD) or Kruskal-Wallis tests among P-AKI and CKD stage 3a (CKD-3a), 3b (CKD-3b) and 4 (CKD-4). Binary data were analyzed using the Chi-square test. A two-sided *p*-value ≤ 0.05 was considered statistically significant.

We used a Cox proportional hazards model, unadjusted and adjusted for age and Charlson Index (modeled as continuous variables), gender, ICU admission and comorbidities: Hypertension, diabetes, coronary artery disease, chronic heart failure, peripheral artery disease and chronic hepatic disease to study in-hospital survival rates. We tested the proportionality assumption of the Cox models using Schoenfeld residual plots. Age and ICU admission were considered as time-dependent covariates. No collinearity was found between the independent variables included in the model. The adjusted Cox model for in-hospital mortality according to AKI severity and baseline renal function was used to create survival curves.

Statistical analysis was carried out using the Statistical Package for Social Sciences software, version 20.0 (SPSS, IBM, Armonk, NY, USA), GraphPad Prism, version 7.04 for Windows (GraphPad Software, La Jolla California USA) and Microsoft Excel 2013 (Microsoft, Inc. Redmond, WA, USA).

## 3. Results

### 3.1. Demographic Characteristics of Patients

We revised all 1584 nephrology consultations and previous or later hospitalizations during the study period; 1269 cases met inclusion criteria (Figure 1), 491 in the P-AKI group and 778 in the AoCKD group.

Characteristics and comparison between groups are shown in Table 2. Individuals in the AoCKD group were older, had higher mean Charlson Index [44] and suffered hypertension, diabetes and cardiovascular disease at a significantly higher rate. Twenty-one patients of the AoCKD (3%) presented a baseline SCr ≥ 4.0 mg/dL. Patients in the P-AKI group were admitted to the ICU more frequently. The distribution across every KDIGO-2012 AKI stage and criterion between P-AKI and AoCKD patients, is shown in Table 2. The proportion of patients who developed AKI stage 1 (ST1) was higher among the AoCKD group; while AKI stage 2 (ST2) was more frequent in the P-AKI group. The criterion used to reach AKI stage 3 (ST3) differed between groups: Most P-AKI patients suffered a ≥ 3.0× increase in SCr from baseline, while the majority of AoCKD patients reached an SCr ≥ 4 mg/dL. The rate of initiation of RRT was similar between groups. In general, we found no statistically significant difference between groups in reaching ST3 (all criteria) (Table 2). We analyzed differences of peak value and SCr net increase (NI)—defined as the difference between peak and baseline SCr values-among P-AKI and AoCKD subjects that fulfilled a specific AKI criterion. Distribution of peak SCr and SCr NI values differed in P-AKI and AoCKD subjects when fulfilling the increment of SCr 1.5–1.9 times baseline criterion (Median peak SCr, P-AKI 1.45 mg/dL vs. AoCKD 2.81 mg/dL, U = 547.5, *p* < 0.001; Median SCr NI, P-AKI 0.57 mg/dL vs. AoCKD 1.2 mg/dL, U = 1045.5, *p* < 0.001), the ST2 criterion (Median peak SCr, P-AKI 2.24 mg/dL vs. AoCKD 3.32 mg/dL, U = 511, *p* < 0.001; Median SCr NI, P-AKI 1.28 mg/dL vs. AoCKD 1.87 mg/dL, U = 921.5, *p* < 0.001), the increment of SCr 3.0 times baseline criterion (Median peak SCr, P-AKI 4.66 mg/dL vs. AoCKD 6.8 mg/dL, U = 8161, *p* < 0.001; Median SCr NI, P-AKI 3.81 mg/dL vs. AoCKD 5.24 mg/dL, U = 10,090.5, *p* < 0.001) or the SCr ≥4.0 mg/dL criterion (Median peak SCr, P-AKI 6.05 mg/dL vs. AoCKD 5.64 mg/dL, U = 23,786, *p* = 0.071; Median SCr NI, P-AKI 5.13 mg/dL vs. AoCKD 3.26 mg/dL, U = 12,110, *p* < 0.001).

### 3.2. In-Hospital Mortality

279 (22%) patients died during hospitalization. We found no statistically significant difference in global mortality rates between groups. More patients died in the ST3 category in both groups compared to the other KDIGO-2012 AKI categories (Table 3).

We studied the distribution of all in-hospital deaths across each KDIGO-2012 AKI criterion in both groups (Table 3). AoCKD presented a higher death rate compared to P-AKI when fulfilling any ST1 criteria or the SCr ≥ 3.0× criterion within ST3 stage. Although the percentage of deaths associated with ST3 was similar between groups, its association with each criterion varied: RRT initiation was the ST3 criterion associated with the highest mortality rate among P-AKI patients, while SCr ≥ 3.0× was the criterion linked to the highest death rate in AoCKD subjects. The percentage of deaths associated with the SCr ≥ 4.0 mg/dL criterion in the P-AKI group was lower when compared to AoCKD subjects (Table 3).

**Figure 2:**
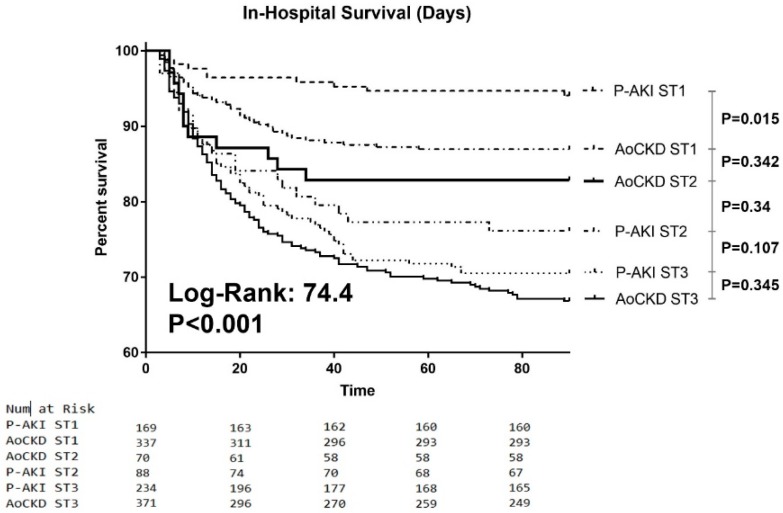
Kaplan-Meier curves. In-hospital survival curves stratified by baseline renal function and AKI severity. P-AKI: Pure Acute kidney injury. AoCKD: Acute-on-chronic kidney disease. AKI: Acute kidney injury. ST1: AKI stage 1. ST2: AKI stage 2. ST3: AKI stage 3.

**Figure 3:**
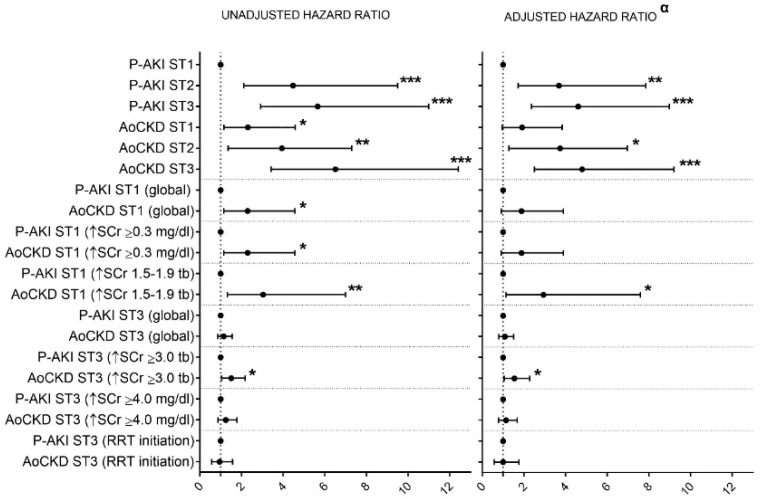
Unadjusted and adjusted hazard ratios (95% confidence interval) for death. P-AKI: Pure acute kidney injury. AoCKD: Acute-on-chronic kidney disease. AKI: Acute kidney injury. ST1: AKI stage 1. ST2: AKI stage 2. ST3: AKI stage 3. SCr: Serum creatinine. Tb: Times baseline. RRT: Renal replacement therapy. α: Models including P-AKI/AoCKD status, age, intensive care unit admission (considered as time-dependent variables), gender, Charlson Index, and comorbidity (hypertension, diabetes, coronary artery disease, chronic heart failure, peripheral arterial disease and chronic hepatic disease). * *p* < 0.05; ** *p* < 0.01; *** *p* < 0.001.

The Cox proportional hazard model, using P-AKI ST1 individuals as the reference group, showed that AoCKD ST3 patients had significantly worse in-hospital survival, with an adjusted hazard ratio (HR) of 4.8 and 95% confidence interval (CI) of 2.5–9.2 (*p* < 0.001), followed by P-AKI ST3 subjects, P-AKI ST2 and AoCKD ST2 patients. In-hospital mortality of those with AoCKD ST1 did not significantly differ from that of P-AKI ST1 individuals (Figure 2 and Figure 3). Other determinants that showed an association to in-hospital mortality were older age (HR: 1.001; 95% CI: 1–1.001; *p* = 0.001), Charlson Index (HR: 1.16; 95% CI: 1.1–1.23; *p* < 0.001), ICU admission (HR: 1.04; 95% CI: 1.03–1.05; *p* < 0.001) and CHF (HR: 1.5; 95% CI: 1.15–1.96; *p* = 0.003).

**Figure 4:**
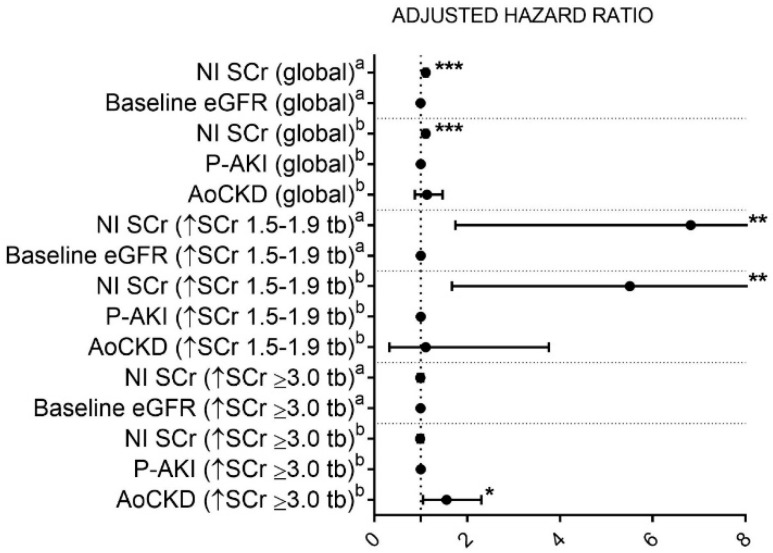
Adjusted hazard ratios (95% confidence interval) for death. NI: Net increase. SCr: Serum creatinine. eGFR: Estimated glomerular filtration rate. P-AKI: Pure acute kidney injury. AoCKD: Acute-on-chronic kidney disease. Tb: Times baseline. a: Models including SCr NI, baseline eGFR, age, Intensive care unit admission (considered as time-dependent variables), gender, Charlson Index, and comorbidity (hypertension, diabetes, coronary artery disease, chronic heart failure, peripheral arterial disease and chronic hepatic disease. b: Models including SCr NI, P-AKI/AoCKD status, age, intensive care unit admission (considered as time-dependent variables), gender, Charlson Index, and comorbidity (hypertension, diabetes, coronary artery disease, chronic heart failure, peripheral arterial disease and chronic hepatic disease. * *p* < 0.05; ** *p* < 0.01; *** *p* < 001.

We also built Cox proportional hazards models to study the effect of fulfilling specific ST1 and ST3 criterion among groups. AoCKD patients presented significantly higher unadjusted mortality HR compared to P-AKI subjects when fulfilling any ST1 stage criterion or the ST3 SCr ≥ 3.0× criterion (Figure 3). After adjustment, only AoCKD individuals who reached ST1 through the SCr ≥ 1.5–1.9× criterion and those that attained ST3 fulfilling the SCr ≥3.0× criterion showed a significantly higher HR for death compared with P-AKI individuals. The AoCKD group showed a small, but not statistically significant, increase in the risk of in-hospital mortality when fulfilling the SCr ≥ 0.3 mg/dL criterion within ST1 or the SCr ≥ 4.0 mg/dL criterion within ST3 (Figure 3). To further investigate the effect of baseline kidney function and its modification during AKI on in-hospital mortality, we added related variables to the analysis. Peak SCr and SCr NI were, as expected, highly correlated variables (*r* = 0.953, *p* < 0.001). SCr NI and baseline eGFR were added to our model as independent variables. Models simultaneously, including baseline eGFR and P-AKI/AoCKD status, were not considered due to multicollinearity. In a global model, including all patients (Figure 4), SCr NI was an independent risk factor for in-hospital mortality, whereas baseline eGFR or P-AKI/AoCKD status were not. This approach was also tested in specific AKI strata that were associated with higher mortality rates among AoCKD patients, namely cases with SCr rise by 1.5–1.9 times or ≥3.0 times baseline (Figure 4). SCr NI proved to be independently associated with mortality among patients that reached an SCr rise 1.5–1.9 times baseline. Conversely, that association was not found among between subjects that suffered an SCr rise ≥ 3.0 times baseline, while in this group, AoCKD status was directly correlated with in-hospital death.

**Figure 5:**
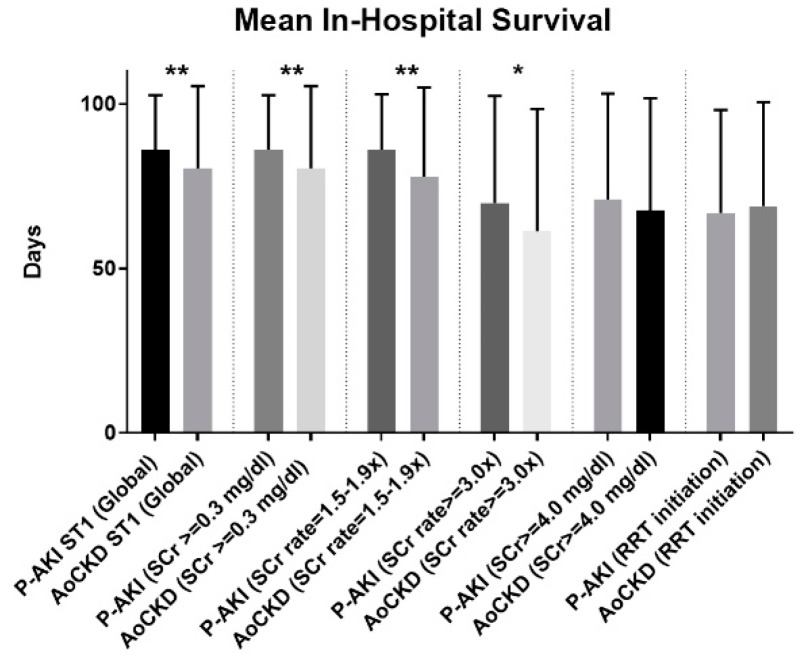
Mean in-hospital survival time (days) by baseline renal function and KDIGO AKI stage and criteria. P-AKI: Pure acute kidney injury. AoCKD: Acute-on-chronic kidney disease. AKI: Acute kidney injury. ST1: AKI stage 1. ST2: AKI stage 2. ST3: AKI stage 3. SCr: Serum creatinine. RRT: Renal replacement therapy. * *p* < 0.05; ** *p* < 0.01.

Mean survival time was significantly higher among ST1 P-AKI individuals when compared to ST1 AoCKD subjects (86.2 ± 16.5 vs. 80.5 ± 24.9 days, *p* < 0.01). ST3 P-AKI patients with an SCr ≥ 3.0× had a better in-hospital survival time compared to AoCKD individuals (69.9 ± 32.5 vs. 61.3 ± 37.1 days, *p* < 0.05) (Figure 5).

### 3.3. Secondary Outcomes

We found no statistically significant difference between groups in need of RRT. The severity of AKI was directly associated with LOS in both groups, but we found no differences in LOS between P-AKI and AoCKD patients. AoCKD group presented a significantly lower time to nephrology consultation compared to P-AKI and a higher dialysis-dependence at discharge (Table 3).

## 4. Discussion

We found that patients with previous impaired renal function were older and had a higher Charlson’s Index, showing that AoCKD individuals differ in their baseline characteristics from the P-AKI group. In addition, we found that the distribution of patients across KDIGO-2012 criteria is different between groups; AKI severity is related with worse short-term outcomes, independently of baseline SCr, and that fulfilling a specific KDIGO-2012 AKI criterion, even within the same AKI stage, is associated with an increased risk of in-hospital death in patients with AoCKD.

In our sample, ST1 was the most common stage reached by CKD-3a and CKD-3b patients, while P-AKI and CKD-4 subjects most frequently reached ST3. The apparent predisposition of individuals with advanced CKD to suffer severe AKI could be due to the increasingly higher baseline SCr associated with CKD-4, which enables reaching the SCr ≥ 4 mg/dL criterion, and thus, ST3, even in the presence of mild insults. A recent study by Hatakeyama et al., [45] also described a higher incidence of ST3 among patients with P-AKI or advanced CKD stages compared to CKD-3a individuals. The percentage of patients in the ST2 stage was surprisingly low in both groups (Table 2). This is not a rare finding; an even lower percentage of patients in the ST2 category was found in individuals from a general (34) and cardiac surgery ICU [46]; the authors propose that this could be explained by the automatic classification of all patients that require RRT as ST3. The path followed by P-AKI and AoCKD individuals to reach a specific AKI stage was also different: Within ST3, the most common criterion fulfilled by AoCKD patients, regardless of CKD stage, was SCr ≥ 4.0 mg/dL, while P-AKI individuals fulfilled more frequently the SCr ≥ 3.0× criterion.

We observed no differences in overall in-hospital mortality rates between groups; but it was significantly higher among AoCKD/ST1 and AoCKD/ST3 subjects compared to P-AKI subjects within that same stage, although only if ST3 was attained through reaching an SCr ≥ 3.0× in the latter group. Peak SCr and SCr NI were significantly higher among AoCKD compared to P-AKI patients in these stages. Lack of differences in mortality between P-AKI and AoCKD individuals for ST2 may be due to the low prevalence of severe CKD among AoCKD subjects—defined as CKD-3b or CKD-4-in this specific stratum. However, within strata associated with higher mortality among AoCKD subjects, such as those with SCr rise by 1.5–1.9 times or ≥3 times vs. baseline, we observed no differences in severe CKD prevalence between AoCKD survivors and non-survivors (1.5–1.9 times baseline, survivors—56.1%, non-survivors—76.9%, *p* = 0.128; three times baseline, survivors—65.3%, non-survivors—56%, *p* = 0.336), indicating a complex relationship between mortality, CKD severity, SCr values and other risk factors among this subpopulation.

When compared with P-AKI/ST1 patients (reference group), patients with AoCKD/ST3 presented the highest adjusted HR for in-hospital death, followed by P-AKI/ST3, P-AKI/ST2 and AoCKD/ST2 patients. We found that AKI severity was associated with higher in-hospital mortality in a stepwise incremental fashion, regardless of baseline renal function; we found no significant differences in adjusted mortality HR within ST3 subjects among those that received RRT or not or those who fulfilled the SCr ≥ 4.0 mg/dL criterion or not. AoCKD patients that reached ST1 or ST3 while fulfilling the SCr ≥ 1.5–1.9× or SCr ≥ 3.0× criteria, respectively, presented a higher adjusted HR of death compared to P-AKI patients. This differential effect on mortality appears among those AoCKD individuals that fulfilled specific AKI criteria that require not a net increase in SCr values, but an increased rate of this parameter with respect to its baseline values. To further support the notion of a differential effect of risk factors, such as AoCKD status or a net increase of SCr in each AKI stratum, we found that SCr NI was closely associated with in-hospital mortality when studying the whole spectrum of AKI. However, if we specifically analyzed the subset of patients that suffered a rise of SCr ≥ 3.0 times baseline, that relationship was no longer found while AoCKD status appeared as an independent risk factor for in-hospital death.

Our results are consistent with previous works: For example, Sawhney et al., [47] observed a higher mortality associated with AKI severity, regardless of baseline eGFR; Zhou et al., [31] reported that AKI severity in patients with decompensated heart failure, assessed using the RIFLE classification, was directly correlated with mortality rates both in P-AKI and AoCKD, but the latter group showed more comorbidities and higher risk of death than P-AKI individuals; Machado et al., [46] also described an increased risk of death associated with AKI severity in patients with preoperatively increased SCr who underwent cardiac surgery. Conversely, higher in-hospital mortality in P-AKI, but not in AoCKD patients, has been linked to AKI severity using the RIFLE and KDIGO-2012 classifications in other studies [35,36,37,38]. Some of these works are based on specific populations, such as the critically ill, describing similar mortality rates in both groups compared to our findings [34,36]. In these settings, the severity of illness could modify the course and outcomes of AKI, explaining the higher mortality observed in P-AKI subjects. Moreover, some of these studies included considerably younger patients in both groups, e.g., Prakash et al., [37]. Our population consists of elderly patients that suffer more frequently relevant comorbid conditions; this could explain the effect of AKI on the excess mortality in AoCKD individuals. We found a similar rate for need of RRT in both groups; irrespective of baseline renal function, these patients showed higher in-hospital mortality (Table 3). Dialysis-dependence at discharge was less frequent in the P-AKI group, regardless of AKI severity. Moreover, approximately 30% of AoCKD were dialysis-dependent at discharge, a lower percentage than that previously described in the literature [32,36], which may be linked to the higher in-hospital mortality observed in this group in our sample. P-AKI patients were admitted more frequently to the ICU; in both groups, AKI severity was directly correlated with admission to the ICU. AKI severity was associated with higher LOS in the P-AKI group, but not in the AoCKD group. This is probably due to AKI severity being intrinsically associated with severity of illness in P-AKI, but not in AoCKD patients.

The present study has several strengths: The study provides a novel approach regarding the influence of each KDIGO-2012 AKI criterion over outcomes in P-AKI and AoCKD patients. All patients have a baseline SCr value to calculate eGFR and the rate of SCr increments; data were obtained prior to the index hospitalization, thus, avoiding the use of surrogate values for calculating baseline renal function. Our cohort consists a heterogeneous sample of hospitalized patients with AKI, not restricted to critically ill or those with a specific condition, such as advanced heart failure, allowing a more reliable representation of a nephrologist day-to-day clinical activity; this increases the generalizability of results not limited to a specific clinical setting. We used standardized and updated definitions of AKI and CKD, following currently available KDIGO guidelines and recorded extensive data on comorbid conditions, which allowed us to adjust for these factors when considering outcomes.

We also acknowledge several important limitations: This is a retrospective single-center study of patients that were treated at least once by the nephrology department, which could lead to a selection bias toward higher AKI severity in both groups, but this circumstance could drive to increased specificity. Extensive efforts were undertaken to adjust for potential confounding, but residual confounding is still possible. Comorbid conditions, such as diabetes, hypertension or coronary artery disease, were considered as dichotomic variables, so there may be residual confounding by severity of these comorbidities. We did not use the urine output criterion. We used only SCr and no other biomarkers for diagnosing and staging AKI. We could not differentiate between true AoCKD and progression of primary renal disease (no biomarkers, few renal biopsies). Follow up was limited to the length of admission to reduce potential bias associated with differential losses to follow up between groups.highlighted.

## 5. Conclusions

To our knowledge, this is the first study that compares the in-hospital outcomes of patients with previous normal and impaired renal function through KDIGO-2012 stages and criteria. The results showed that AKI KDIGO-2012 classification predicted in-hospital mortality in both P-AKI and AoCKD patients, but we found a differential effect of AKI KDIGO-2012 criteria on outcomes among AoCKD patients compared to P-AKI subjects. Several authors have proposed that certain specific conditions, such as CKD or advanced age, should be taken into account while applying AKI classifications [35,48]. RIFLE, AKIN and/or KDIGO-2012 were designed for and tested in patients with previously normal renal function, but considering the different outcomes observed among P-AKI and AoCKD subjects within the same AKI stage and criterion maybe one size does not fit all AKI patients. We consider that AoCKD may constitute a separate clinical syndrome, and due to the increasing prevalence of CKD, the development of adequate staging criteria for AoCKD could help prompt the diagnosis and administration of appropriate therapy.

## Figures and Tables

**Table 1:** KDIGO-2012 acute kidney injury (AKI) classification and criteria.

Stage	Serum Creatinine	Urine Output
1	1.5–1.9 times baseline	<0.5 mL/kg/h for 6–12 h
	OR	
	≥0.3 mg/dL increase	
2	2.0–2.9 times baseline	<0.5 mL/kg/h for ≥12 h
3	3.0 times baseline	<0.3 mL/kg/h for ≥24 h
	OR	OR
	Increase in serum creatinine to ≥4.0 mg/dL	Anuria for ≥12 h to ≥4.0 mg/dL
	OR	
	Initiation of renal replacement therapy	
	OR	
	In patients <18 years. Decrease in eGFR to <35 mL/min/1.73 m^2^	

**Table 2:** Baseline Characteristics of AKI and AoCKD Groups and Distribution across KDIGO-2012 Criteria.

	ALL	P-AKI			AoCKD			*p* Value^2^
			AoCKD	*p* Value^1^				
					CKD-3A	CKD-3B	CKD-4	
N	1269	491	778		221	282	275	
**Male sex—No. (%)**	883 (70)	339 (69)	544 (70)	0.739	162 (73)	198 (70)	184 (67)	0.476
**Age (years)—Median (IQR)**	75 (65–81)	71 (61–79)	77 (69–83)	<0.001	76 (69–82)	78 (70–83)	77 (67–83)	<0.001
**HTN—No. (%)**	1125 (89)	395 (80)	730 (94)	<0.001	206 (93)	268 (95)	256 (93)	<0.001
**DM—No. (%)**	536 (42)	158 (32)	378 (49)	<0.001	96 (43)	145 (51)	137 (50)	<0.001
**CAD—No. (%)**	385 (30)	107 (22)	278 (36)	<0.001	73 (33)	105 (37)	100 (36)	<0.001
**CHF—No. (%)**	491 (39)	140 (29)	351 (45)	<0.001	94 (43)	146 (52)	111 (40)	<0.001
**PAD—No. (%)**	392 (31)	108 (22)	284 (37)	<0.001	89 (40)	112 (40)	83 (30)	<0.001
**CHD—No. (%)**	231 (18)	37 (8)	36 (5)	0.03	11 (5)	14 (5)	11 (4)	0.171
**Charlson Comorbidity Index (SD)**	4 (3–6)	4 (2–6)	5 (3–6)	<0.001	4 (3–6)	5 (3–6)	6 (4–7)	<0.001
**Unit of Admission**								
**Medical Unit—No. (%)**	808 (64)	180 (37)	281 (36)	0.845	91 (41)	110 (39)	80 (29)	0.025
**IC** **U—No. (%)**	241 (19)	117 (24)	124 (16)	<0.001	44 (20)	42 (15)	38 (14)	0.001
**AKI Type**								
**Community Acquired—No. (%)**	870 (69)	160 (33)	239 (31)	0.485	74 (34)	90 (32)	75 (27)	0.396
**KDIGO-2012 AKI Stage 1 (global)—No. (%)**	506 (40)	169 (34)	337 (43)	0.002	115 (53)	145 (51)	77 (28)	<0.001
**≥0.3 mg/dL**	506 (40)	169 (34)	337 (43)	0.002	115 (53)	145 (51)	77 (28)	<0.001
**SCr 1.5–1.9×**	264 (21)	115 (23)	149 (19)	0.068	59 (27)	70 (25)	20 (7)	<0.001
**KDIGO-2012 AKI Stage 2 (SCr 2.0–2.9×)—No. (%)**	158 (13)	88 (18)	70 (9)	<0.001	42 (19)	27 (10)	1 (0.4)	<0.001
**KDIGO-2012 AKI Stage 3 (global)—No. (%)**	605 (48)	234 (48)	371 (48)	0.992	60 (28)	112 (39)	199 (72)	<0.001
**SCr ≥ 3.0×**	354 (28)	229 (47)	125 (16)	<0.001	46 (21)	54 (19)	25 (9)	<0.001
**SCr ≥ 4.0 mg/dL**	503 (40)	150 (31)	353 (45)	<0.001	48 (22)	108 (38)	197 (71)	<0.001
**Initiation RRT**	167 (13)	62 (13)	105 (14)	0.656	16 (7)	24 (9)	65 (24)	<0.001
**Baseline SCr (mg/d** **L)**	1.4 (1–2)	0.9 (0.7–1.1)	1.9 (1.5–2.5)	<0.001	1.4 (1.2–1.5)	1.8 (1.6–2)	2.7 (2.4–3.2)	<0.001
**Peak SCr (mg/d** **L)**	3.4 (2.2–5.2)	2.5 (1.5–4.5)	3.7 (2.6–5.5)	<0.001	2.6 (2–3.8)	3.4 (2.6–4.9)	5 (3.9–6.7)	<0.001
**SCr Net Increase (mg/dL)**	1.6 (0.7–3.3)	1.6 (0.6–3.6)	1.7 (0.9–3)	0.232	1.2 (0.7–2.4)	1.6 (0.8–3.2)	2.2 (1.2–3.5)	<0.001
**Discharge SCr (mg/d** **L)**	1.9 (1.3–2.9)	1.2 (0.9–1.7)	2.4 (1.7–3.5)	<0.001	1.7 (1.4–2.2)	2.3 (1.8–2.8)	3.6 (2.7–4.9)	<0.001

Data are expressed as mean ± standard deviation (SD), median and interquartilic range (IQR) or number (percentage). P-AKI, pure acute kidney injury; AoCKD, acute on chronic kidney disease; HTN, Hypertension; DM, diabetes mellitus; CAD, coronary artery disease; CHF, chronic heart failure; PAD, peripheral arterial disease; CHD, chronic hepatic disease; ICU, intensive care unit; AKI, acute kidney injury; SCr, serum creatinine; RRT, renal replacement therapy. *p* Value^1^: Comparison of P-AKI vs. AoCKD (all patients). *p* Value^2^: Comparison of P-AKI vs. AoCKD stages.

**Table 3:** Primary and secondary endpoints. Mortality rates of subjects that met each KDIGO stage/criterion within P-AKI/AoCKD groups.

	ARF	AoCKD	*p* Value
N	491	778	
**Primary Endpoint**			
In-Hospital Mortality—No. (%)	100 (20.4)	179 (23)	0.15
**Secondary Endpoints**			
Initiation of RRT—No. (%)	62 (12.6)	105 (13.5)	0.36
Length of Hospital Stay (days)	12 (7–25)	12 (7–21)	0.08
Time to Nephrology Consultation (days)	4 (1–8)	3 (1–6)	<0.001
Dialysis Dependence at Discharge—No. (%)	7 (1.4)	40 (5.1)	<0.001
**In-Hospital Mortality**	**ARF**	**AoCKD**	***p* Value**
KDIGO-2012 AKI Stage 1 (global)	10 (5.9)	44 (13.1)	0.014
≥0.3 mg/dL	10 (5.9)	44 (13.1)	0.014
SCr 1.5–1.9×	7 (6.1)	26 (17.4)	0.006
KDIGO-2012 AKI Stage 2	21 (23.9)	12 (17.1)	0.302
KDIGO-2012 AKI Stage 3 (global)	69 (29.5)	123 (33.2)	0.345
SCr 3.0×	67 (29.3)	50 (40)	0.04
SCr ≥ 4.0 mg/dL	41 (27.3)	116 (32.9)	0.221
Initiation RRT	24 (38.7)	39 (37.1)	0.84

Data are expressed as mean ± SD or number (percentage). P-AKI: Pure acute kidney injury; AoCKD: Acute on chronic kidney disease; RRT: Renal replacement therapy; AKI: Acute kidney injury; SCr: Serum creatinine.
